# Antibiotic Stress Reshapes Physiological Homeostasis, Lipid Metabolism, and Culture-Associated Bacterial Communities in *Chlorella pyrenoidosa*

**DOI:** 10.3390/ijms27188325

**Published:** 2026-09-18

**Authors:** Linyinxue Dong, Yanxin Zhang, Aloysius Wong, Meihao Huang, Liangliang Mu, Aike Yi, Xuxiong Huang

**Affiliations:** 1 College of Fisheries and Life Science, Shanghai Ocean University, Shanghai 201306, China; donglinyinxue@wku.edu.cn (L.D.); m230100235@st.shou.edu.cn (Y.Z.); m240100264@st.shou.edu.cn (M.H.); 2022000044@jou.edu.cn (L.M.); m240100277@st.shou.edu.cn (A.Y.); 2 College of Science, Mathematics and Technology, Wenzhou-Kean University, Wenzhou 325060, China; alwong@kean.edu; 3 Wenzhou Municipal Key Laboratory for Applied Biomedical and Biopharmaceutical Informatics, Wenzhou-Kean University, Wenzhou 325060, China; 4 School of Marine Science and Fisheries, Jiangsu Ocean University, Lianyungang 222006, China; 5 China-ASEAN Belt and Road Joint Laboratory on Mariculture Technology (Shanghai), Shanghai Ocean University, Shanghai 201306, China; 6 National Demonstration Center for Experimental Fisheries Science Education, Shanghai Ocean University, Shanghai 201306, China

**Keywords:** *Chlorella pyrenoidosa*, antibiotic stress, algal physiology, culture-associated bacteria, lipid metabolism, untargeted metabolomics, microalgae–bacteria interactions

## Abstract

Antibiotics are widely used to decontaminate microalgal cultures, but their effects on algal physiology and associated bacterial communities remain unclear. We examined the concentration- and compound-dependent responses of *Chlorella pyrenoidosa* to ampicillin sodium, sulfanilamide, chloramphenicol, and streptomycin sulfate. Growth, culturable bacterial abundance, pigment content, photosynthetic performance, and fatty-acid composition were assessed across a mixed-antibiotic concentration gradient and under individual treatments. Higher antibiotic concentrations suppressed bacteria but inhibited algal growth, reduced chlorophyll accumulation, and altered fatty-acid composition. Physiological disruption increased in the order ampicillin sodium < sulfanilamide < chloramphenicol < streptomycin sulfate. Streptomycin sulfate produced the strongest response, reducing maximum quantum yield of PSII photochemistry (Fv/Fm) and light-use efficiency by 51.4% and 44.4%, respectively, while increasing total polyunsaturated fatty acids from 59.68% to 79.57%. 16S rRNA gene sequencing and untargeted metabolomics showed that streptomycin sulfate reduced bacterial diversity and increased the relative abundance of Proteobacteria from 44.54% to 90.66%. Lipid-related pathways were altered, particularly α-linolenic acid and glycerophospholipid metabolism. Bacterial-community and metabolomic changes covaried under STRS exposure, and responsive taxa correlated with phospholipid- and oxylipin-related metabolites. These findings identify antibiotic acts as a selective stressor that alters algal physiology, lipid metabolism, and associated bacterial communities, highlighting the need for species-specific decontamination strategies.

## 1. Introduction

Microalgae are major primary producers in aquatic ecosystems and contribute to carbon fixation, nutrient cycling, and the support of aquatic food webs [1]. Beyond their ecological significance, microalgae have been widely used in biofuels, food and feed, nutraceuticals, wastewater treatment, and the production of high-value bioproducts [2]. Among commercially relevant species, *Chlorella pyrenoidosa* (*C. pyrenoidosa*) has attracted considerable interest because it can grow under autotrophic, mixotrophic, and heterotrophic conditions. Mixotrophic and heterotrophic cultivation can support high biomass productivity and facilitate large-scale production [3]. The resulting biomass is rich in proteins, essential amino acids, and unsaturated fatty acids [4]. In addition, its genetic background is increasingly well characterized, and genetic tools for Chlorella are advancing [5]. Together, these features make *C. pyrenoidosa* suitable for both biotechnological production and mechanistic studies of algal physiology.

In both fundamental research and industrial applications, the establishment of axenic microalgal cultures is essential. It provides a controlled system for studying algal physiology, molecular biology, and genetic engineering, as well as quality control and product consistency in high-value applications, especially when the biomass or extracted compounds are intended for pharmaceutical, food, or other sensitive uses [6]. This need is especially acute under mixotrophic and heterotrophic conditions, where added organic carbon can also support the proliferation of contaminating microorganisms [7]. However, axenic practice involves a basic assumption that is not always examined: a treatment that efficiently removes bacteria is also physiologically acceptable to the alga.

Antibiotic treatment remains the most practical and widely used approach for establishing axenic microalgal cultures, because it is comparatively inexpensive, technically accessible, and readily implemented in most cases [8]. Antibiotics can be broadly classified as bactericidal or bacteriostatic according to their effects on susceptible bacteria [9]. Bactericidal antibiotics, such as penicillin and streptomycin, kill susceptible bacteria, whereas bacteriostatic antibiotics, such as sulfanilamide and chloramphenicol, inhibit bacterial growth and proliferation without necessarily causing cell death [10,11,12]. These effects are achieved through diverse mechanisms, including disruption of cell-wall synthesis and inhibition of protein synthesis or other essential cellular processes, which may alter algal physiology, biochemical composition, and bacterial interactions at the same time.

Despite the routine use of antibiotics for microalgal axenization and decontamination, treatment success is often assessed mainly by the extent of bacterial removal. The physiological cost to the target alga has received much less attention, particularly when high antibiotic concentrations or antibiotic mixtures are used. Moreover, few studies have examined algal physiology, fatty-acid composition, bacterial community structure, and metabolic responses within the same experimental framework. It therefore remains unclear whether antibiotic-induced physiological changes occur independently or coincide with the restructuring of culture-associated bacterial communities.

To address these questions, we investigated the responses of *C. pyrenoidosa* to four commonly used antibiotics: ampicillin sodium (AMPS), sulfanilamide (SA), chloramphenicol (CHL), and streptomycin sulfate (STRS) at three connected levels. First, the effects of a mixed-antibiotic concentration gradient on growth, pigment composition, and fatty-acid profiles were evaluated. Second, antibiotics with distinct cellular targets were compared to determine their individual effects. Finally, the antibiotic that produced the strongest physiological response was examined in greater depth through bacterial community profiling, metabolomics, and integrative association analyses. We asked whether antibiotic-based decontamination acts as a simple bacterial removal step or as a selective ecological disturbance that reshapes both the algal host and its associated microbiome. This study aims to deepen our understanding of how *C. pyrenoidosa* responds to antibiotic-induced stress, contributing to both theoretical research on algal physiology and practical applications in microalgal biotechnology.

## 2. Results

### 2.1. Effects of Antibiotics Concentration on C. pyrenoidosa

The control group (0 µg/mL) exhibited optimal growth, immediately entering the exponential growth phase and reaching the highest cell density of 17.2 × 10^6^ cells/mL by day 5, demonstrating the absence of growth inhibition under antibiotic-free conditions. In contrast, the treatment groups displayed a delayed growth pattern, with slower growth on day 1, followed by an exponential phase from day 2 onward.

At per-antibiotic concentrations of 50 µg/mL (MIX-50; 200 µg/mL total) and 100 µg/mL (MIX-100; 400 µg/mL total), *C. pyrenoidosa* showed moderate growth inhibition, with final cell densities of 13.6 × 10^6^ and 12.9 × 10^6^ cells/mL, respectively. At 200 and 400 µg/mL per antibiotic, growth suppression became more pronounced, and the final cell densities decreased to 12.4 × 10^6^ and 10.2 × 10^6^ cells/mL, respectively. The most severe inhibition occurred in the MIX-800 group, in which cell density remained significantly lower throughout the experiment and reached only 6.3 × 10^6^ cells/mL by day 5, a 63.4% reduction relative to the control (*p* < 0.05).

By the end of the experiment, the relative growth rate in the control group was the highest, measuring 2.05 times that of the MIX-800 group (Table 1). Statistical analysis revealed that the relative growth rate in the MIX-50 group did not differ significantly from the MIX-100 and MIX-200 groups (*p* > 0.05) but was significantly higher than that of the MIX-400 and MIX-800 groups (*p* < 0.05).

**Figure 1:**
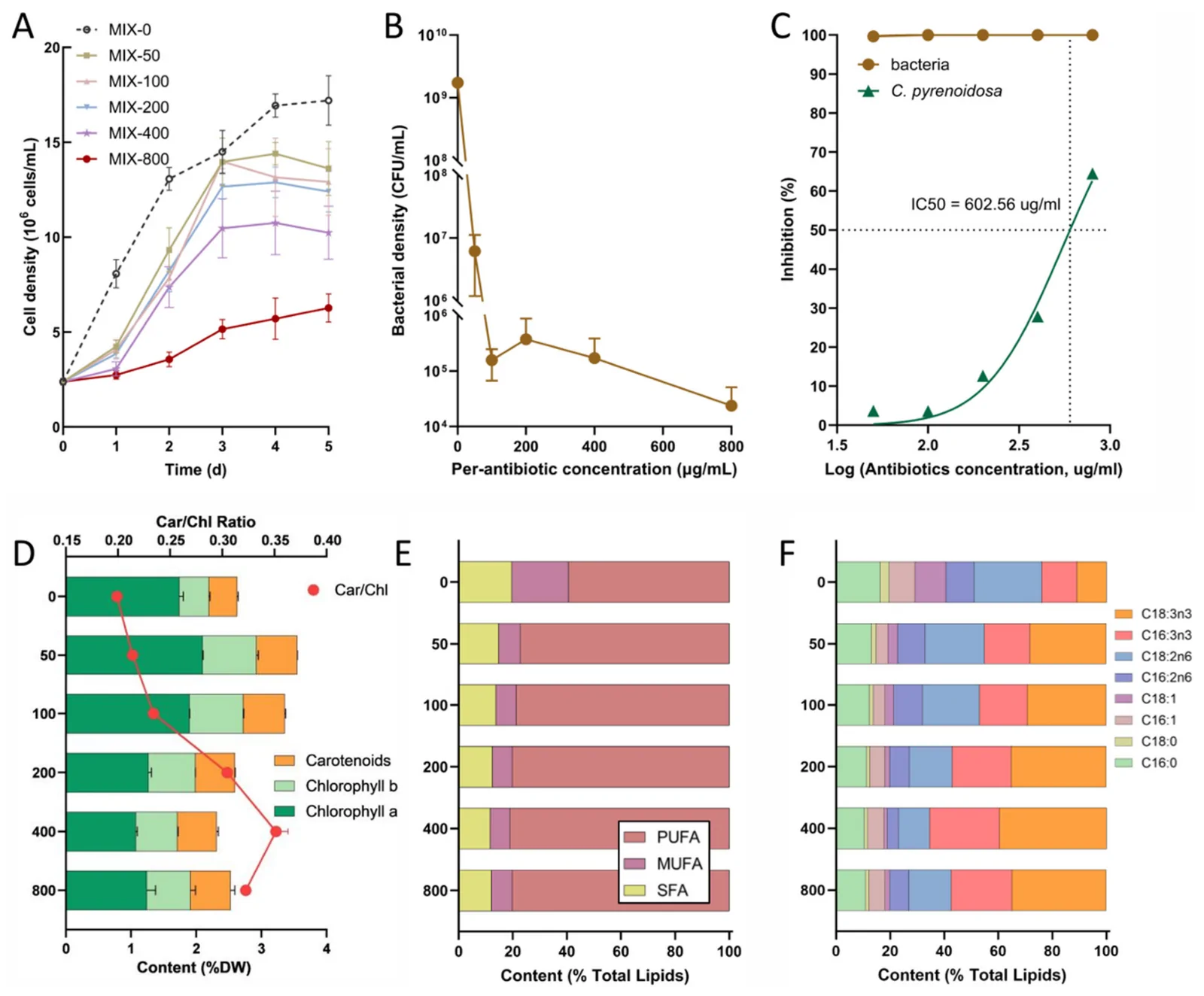
Effects of mixed-antibiotic concentration on *Chlorella pyrenoidosa*. (**A**) Growth curves based on cell density (×10^6^ cells/mL); (**B**) culturable bacterial abundance in the culture medium; (**C**) concentration–response curve for inhibition of cell density over 72 h; (**D**) pigment composition; (**E**) lipid-class composition; and (**F**) individual fatty-acid profiles on day 5. Per-antibiotic concentration refers to the concentration of each antibiotic in the four-antibiotic mixture.

Mixed-antibiotic treatment markedly reduced culturable bacterial abundance, as determined by plate counting (Figure 1B). At the lowest concentration (MIX-50), culturable bacterial abundance decreased significantly to 6.17 × 10^6^ CFU/mL, representing a 99.64% reduction relative to the control (1.74 × 10^9^ CFU/mL). As the per-antibiotic concentration increased to 400 and 800 µg/mL, culturable bacterial abundance decreased to 1.71 × 10^5^ and 2.38 × 10^4^ CFU/mL, respectively, representing reductions of more than 99.99% relative to the control (*p* < 0.05).

*C. pyrenoidosa* was more tolerant of the antibiotic mixture than the culturable bacteria, as illustrated by the concentration-dependent inhibition of algal cell density over 72 in Figure 1C. The IC_50_ was 602.56 µg/mL on a per-antibiotic basis, equivalent to a total nominal antibiotic concentration of 2410.24 µg/mL. At low per-antibiotic concentrations (<200 µg/mL), inhibition remained below 10%, suggesting a limited impact on algal growth. However, inhibition increased sharply above 400 µg/mL and exceeded 60% at 800 µg/mL, indicating severe toxicity.

Chlorophyll content exhibited a biphasic response to antibiotic concentration (Figure 1D). At low concentrations, pigment accumulation was enhanced, with chlorophyll *a*, chlorophyll *b*, and carotenoids all exceeding the control level. Total pigment content increased from 2.63% dry weight (DW) in the control to 3.55% and 3.36% DW at 50 and 100 µg/mL, respectively. However, from 200 µg/mL onward, chlorophyll content declined markedly, particularly chlorophyll *a*, which decreased to 1.27% and 1.07% DW at 200 and 400 µg/mL, respectively. In contrast, carotenoid content remained relatively stable (approximately 0.61% DW) across antibiotic treatments (*p* > 0.05) but consistently exceeded the control level (*p* < 0.05). As a result, the carotenoid-to-chlorophyll ratio increased progressively with concentration, from 0.20 in the control to 0.30–0.35 at 200–400 µg/mL, indicating a shift in pigment allocation from light-harvesting chlorophylls toward relatively greater photoprotective carotenoid retention under elevated antibiotic stress.

Mixed-antibiotic concentration markedly altered the fatty-acid composition of *C. pyrenoidosa* (Figure 1E). Compared with the control, the proportion of polyunsaturated fatty acids (PUFA) increased sharply from 59.35% of total lipids to 77.11–81.03% in the antibiotic-treated groups, whereas saturated fatty acids (SFA) decreased from 19.69% to 11.74–14.89% and monounsaturated fatty acids (MUFA) decreased from 20.95% to 7.22–7.98%. The highest PUFA proportion was observed at 400 µg/mL (81.03%), indicating that mixed-antibiotic exposure promoted a pronounced shift in lipid composition toward a more unsaturated state.

At the individual fatty-acid level (Figure 1F), antibiotic treatment reduced the proportions of C16:0 (palmitic acid), C18:0 (stearic acid), and C18:1 (oleic acid), while strongly increasing n-3 PUFA species, particularly C16:3n3 and C18:3n3 (α-linolenic acid; ALA). C18:3n3 increased from 10.84% in the control to 28.21%, 29.19%, 35.16%, 39.54%, and 34.91% at 50, 100, 200, 400, and 800 µg/mL, respectively, while C16:3n3 increased from 13.11% to 16.89–25.90% across treatments. In contrast, C18:2n6 (linoleic acid) declined progressively with increasing concentration, especially at 200–400 µg/mL. These results indicate that the PUFA enrichment under antibiotic stress was driven primarily by accumulation of ALA.

Overall, increasing antibiotic concentrations progressively suppressed culturable bacteria, caused moderate algal growth inhibition at low concentrations, and triggered a physiological transition characterized by chlorophyll loss and PUFA enrichment at high concentrations.

### 2.2. Effects of Antibiotics Type on C. pyrenoidosa

**Figure 2:**
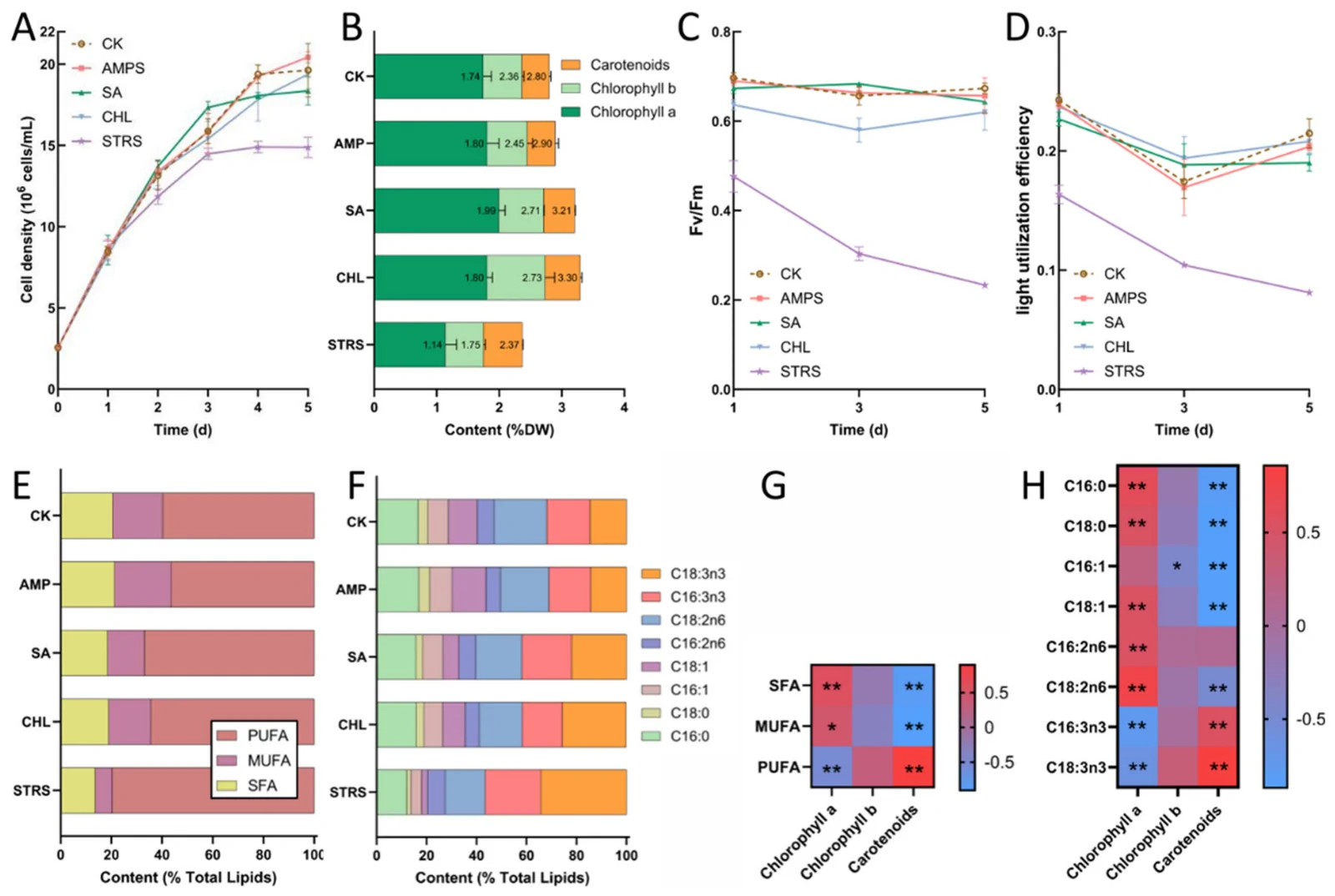
Effects of individual antibiotics on *Chlorella pyrenoidosa*. (**A**) Growth curves based on cell density (×10^6^ cells/mL); (**B**) pigment contents (% DW) on day 5; (**C**) Fv/Fm (the maximum quantum yield of photosystem II photochemistry) and (**D**) α (light-use efficiency) during cultivation; (**E**) lipid-class composition and (**F**) individual fatty-acid profiles on day 5; and correlations between pigment composition and (**G**) lipid classes and (**H**) individual fatty acids. Statistical significance is indicated by * for *p* < 0.05 and ** for *p* < 0.01.

The effects of individual antibiotics on the growth of *C. pyrenoidosa* differed markedly (Figure 2A and Table 2). AMPS and CHL showed little overall inhibitory effect, with growth curves and relative growth rates largely comparable to the control. SA caused a slight reduction in proliferation, with a relative growth rate of 0.44 ± 0.01 compared with 0.46 ± 0.01 in the control. In contrast, STRS exhibited the strongest inhibitory effect, resulting in the lowest cell density from day 2 onward and a markedly reduced final cell density on day 5 (14.88 × 10^6^ cells/mL). Consistently, STRS produced the lowest relative growth rate (0.38 ± 0.00; *p* < 0.05). Specifically, cell density in the STRS-treated group decreased by 24.18%, and growth rate by 17.39%, relative to CK. In contrast, the AMPS, SA, and CHL groups showed no significant differences from CK (*p* > 0.05), indicating that *C. pyrenoidosa* was substantially more sensitive to STRS than to the other antibiotics tested.

Single antibiotics differentially affected pigment accumulation in *C. pyrenoidosa* on day 5 (Figure 2B). Compared with the control, AMPS caused only minor changes in chlorophyll *a*, chlorophyll *b*, carotenoids, and total pigment content. In contrast, SA and CHL increased total pigment content to 3.21% and 3.30% DW, respectively, compared with 2.80% DW in the control. The CHL treatment showed the highest chlorophyll *b* and carotenoid contents among all groups. STRS produced a distinct response, characterized by a marked decrease in chlorophyll *a* to 1.14% DW and a reduction in total pigment content to 2.37% DW, despite a higher carotenoid content than the control. These results indicate that the tested antibiotics induced distinct pigment-response patterns, with STRS exerting the strongest disruptive effect on the photosynthetic pigment system.

Additionally, the effects of different antibiotics on photosynthetic performance were evaluated including the maximum quantum yield of photosystem II photochemistry (Fv/Fm; Figure 2C), light-use efficiency (α; Figure 2D), effective quantum yield of PSII [Y(II); Figure S1], and maximum electron transport rate (ETRmax; Figure S2). AMPS and SA had little effect on Fv/Fm and α relative to the control, while CHL caused only moderate suppression. In contrast, the STRS group exhibited a significant and progressive decline in photosynthetic performance, with Fv/Fm decreasing to 0.34 ± 0.03 (51.4% lower than the control, *p* < 0.05) and α dropping to 0.135 ± 0.011 (44.4% lower than the control, *p* < 0.05), indicating severe impairment of PSII activity and light utilization efficiency.

Antibiotic-treated groups showed varying effects on lipid composition (Figure 2E). The AMPS, SA, and CHL groups exhibited relatively stable PUFA levels, ranging from 56.28% to 66.92%, with no significant differences compared to CK (*p* > 0.05). However, the STRS group exhibited a significant increase in PUFA content, reaching 79.57 ± 0.62%, which was 33.33% higher than the control (59.68 ± 1.11%) (*p* < 0.05).

A detailed analysis of fatty acid composition (Figure 2F) revealed that the increase in PUFAs in the STRS group was primarily attributed to a rise in n-3 PUFAs, particularly C16:3n3 and C18:3n3. The levels of C16:3n3 and C18:3n3 in the STRS group were significantly higher than those in the CK group and all other antibiotic treatments (*p* < 0.05), with increases of 28.5% and 135.0%, respectively, compared to CK.

Correlations between pigments and fatty acids, calculated across the single- and mixed-antibiotic treatments, are presented in Figure 2G,H. Chlorophyll *a* exhibited a highly significant positive correlation (*p* < 0.01) with C16:0 (r = 0.620), C16:2n6 (r = 0.525), C18:0 (r = 0.518), C18:1 (r = 0.519), C18:2n6 (r = 0.751), and total SFAs (r = 0.620). Conversely, chlorophyll *a* was strongly negatively correlated (*p* < 0.01) with C16:3n3 (*r* = −0.772), C18:3n3 (*r* = −0.624), and total PUFAs (*r* = −0.514). Carotenoids exhibited an inverse trend, showing a strong negative correlation with SFAs (C16:0, *r* = −0.822; C18:0, *r* = −0.866; *p* < 0.01) and MUFAs (C16:1, *r* = −0.868; C18:1, *r* = −0.867; *p* < 0.01), but a highly significant positive correlation with PUFAs (*r* = 0.898, *p* < 0.01), particularly C18:3n3 (*r* = 0.858, *p* < 0.01).

### 2.3. Effect of STRS on the Culture-Associated Bacterial Community

In the antibiotic-type screening, AMPS, SA, and CHL produced relatively mild effects, whereas STRS caused the strongest inhibitory phenotype. STRS was therefore selected for in-depth bacterial community analysis, due to its severe inhibition of algal growth and photosynthesis, drastic lipid remodeling, and therefore, maybe potential major microbial community shifts.

**Figure 3:**
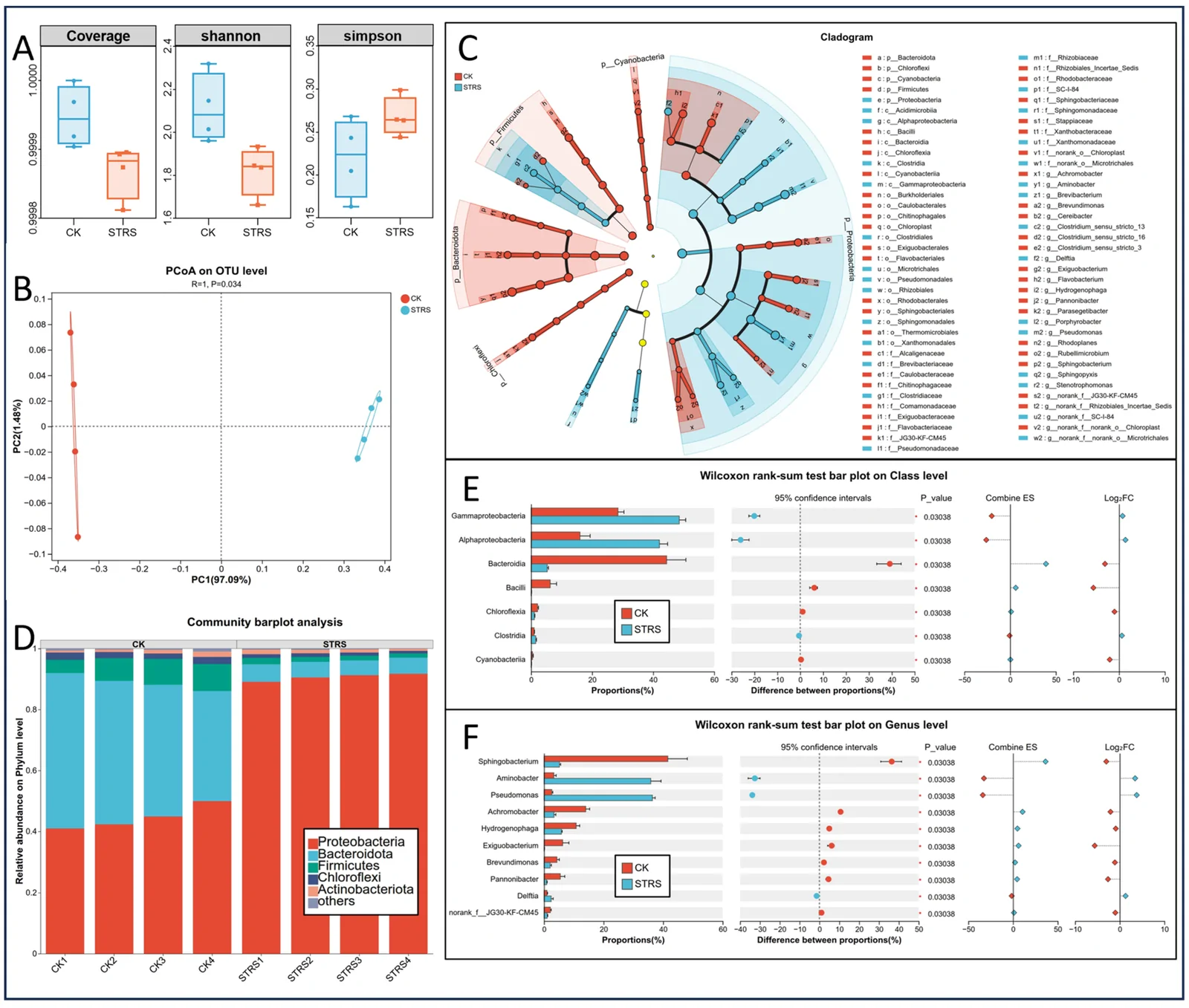
Effects of STRS on the diversity and taxonomic composition of the bacterial community associated with *Chlorella pyrenoidosa* cultures. (**A**) Good’s coverage, Shannon diversity, and Simpson dominance; (**B**) principal coordinates analysis based on Bray–Curtis dissimilarities at the OTU level; (**C**) LEfSe cladogram of differentially abundant bacterial lineages; (**D**) relative abundance of bacterial phyla; and Wilcoxon rank-sum comparisons at the (**E**) class level and (**F**) genus level.

A total of 307,652 high-quality sequences with an average length of 419 bp were obtained. Good’s coverage exceeded 0.99 for all samples, indicating adequate sequencing depth for subsequent analyses (Figure 3A; Table S2). PCoA based on Bray–Curtis dissimilarities at the OTU level showed clear separation between the CK and STRS groups; PC1 and PC2 explained 97.09% and 1.48% of the total variation, respectively (Figure 3B; Table S3).

After STRS treatment, the bacterial community had a lower Shannon index and a higher Simpson dominance index, indicating lower diversity and greater taxonomic dominance under STRS exposure (Figure 3A; Table S3). These results suggest that STRS acted as a strong ecological filter, reducing community complexity and favoring a narrower subset of surviving taxa.

Differences in bacterial lineages between treatments were visualized using a LEfSe cladogram (Figure 3C), which showed clear phylogenetic differentiation between the bacterial communities associated with the CK and STRS cultures. STRS treatment altered microbial composition at multiple taxonomic levels, including phylum, class, order, family, and genus. At the phylum level, STRS caused a pronounced shift toward Proteobacteria dominance, whose relative abundance increased from 44.54% in CK to 90.66% in STRS, accompanied by marked depletion of several non-proteobacterial lineages (Figure 3D).

This taxonomic restructuring was further supported by Wilcoxon rank-sum analysis (Table S5). At the class level (Figure 3E), the increased abundance of Gammaproteobacteria and Alphaproteobacteria was the primary driver of Proteobacteria enrichment. At the order level (Figure S3), Rhizobiales and Pseudomonadales increased from 11.23% to 38.50% and from 2.61% to 36.43%, respectively. In contrast, Sphingobacteriales and Burkholderiales declined. These changes were also reflected at family levels (Figure S4). Rhizobiaceae and Pseudomonadaceae increased markedly in the STRS group, while Sphingobacteriaceae showed the largest decrease. At the genus level (Figure 3F), *Aminobacter*, *Pseudomonas* and *Delftia* were strongly enriched after STRS exposure, whereas *Sphingobacterium* declined substantially. These results indicate that STRS selectively restructured the bacterial community by favoring specific proteobacterial lineages.

### 2.4. Integrated Analysis of Metabolomics and Microbiome

**Figure 4:**
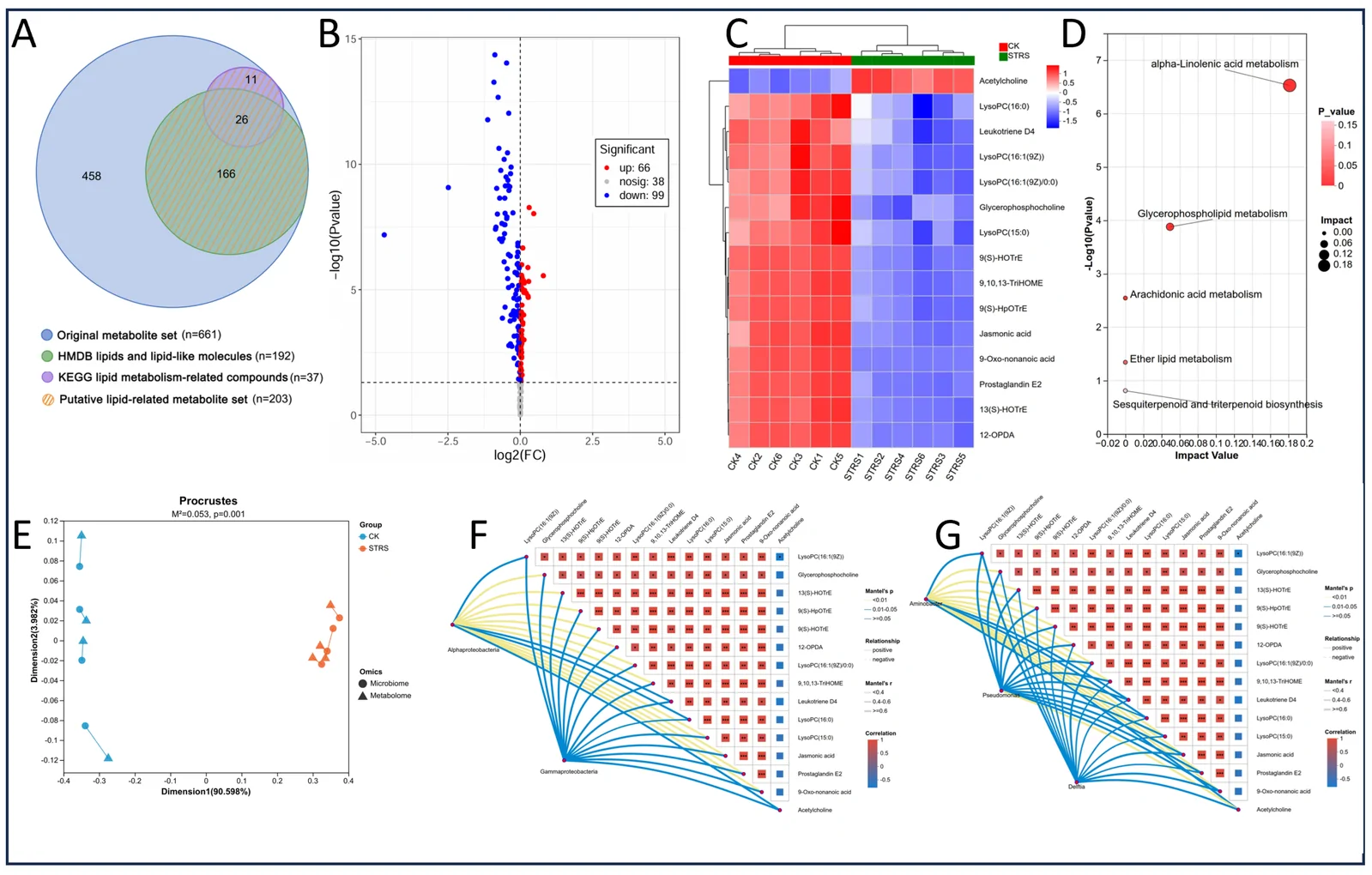
STRS-induced remodeling of lipid-related metabolites and its association with bacterial community composition. (**A**) Identification of the putative lipid-related metabolite set; (**B**) volcano plot of differential lipid-related metabolites; (**C**) hierarchical-clustering heatmap of the 15 representative differential lipid-related metabolites; (**D**) KEGG pathway enrichment and topology analysis; (**E**) Procrustes analysis showing significant concordance between bacterial community composition and metabolomic profiles; and Mantel-test network heatmaps showing associations of key lipid-related metabolites with bacterial classes (**F**) and genera (**G**). Statistical significance is indicated by * for *p* < 0.05, ** for *p* < 0.01, and *** for *p* < 0.001.

Untargeted metabolic analysis of *C. pyrenoidosa* after STRS treatment detected 8583 valid peaks. Among the 661 annotated metabolites, 192 were classified as lipids and lipid-like molecules in HMDB, while 37 were assigned to lipid metabolism-related pathways in KEGG. After removing duplicates, 203 metabolites were generated as the putative lipid-related metabolite set to be further analyzed (Figure 4A; Table S6).

Differential analysis identified substantial changes in the lipid-related metabolite set (Figure 4B). Compared with CK, 66 metabolites increased and 99 decreased after STRS treatment. Hierarchical clustering clearly separated the CK and STRS samples (Figure 4C). Most representative metabolites were less abundant in the STRS group, including several lysophosphatidylcholines, glycerophosphocholine, and oxylipin-related metabolites. The topology analyses of KEGG enrichment identified ALA metabolism as the most strongly affected pathway, with the highest pathway-impact value (Figure 4D). This result was consistent with the significant enrichment in ALA portion shown in Figure 2F.

Bacteria and microalgae interact in complex and dynamic ways. To further investigate the linkage between microbial community restructuring and metabolic reprogramming under STRS exposure, integrated analysis of the microbiome and metabolome was performed. Procrustes analysis (Figure 4E) showed significant concordance between bacterial community composition and metabolite abundance patterns (M^2^ = 0.053, *p* = 0.001).

Mantel tests provided additional evidence that changes in bacterial community composition under STRS exposure were associated with alterations in the lipid-related metabolite profile of *C. pyrenoidosa*. At the class level, Alphaproteobacteria showed particularly strong associations with key phospholipid- and oxylipin-related metabolites, whereas Gammaproteobacteria displayed a distinct association pattern (Figure 4F). At the genus level, *Aminobacter* showed the strongest and most widespread associations with the selected metabolites, followed by *Pseudomonas* and *Delftia* (Figure 4G). These results indicated that STRS-induced bacterial community restructuring occurred in parallel with extensive remodeling of the lipid-related metabolome.

## 3. Discussion

### 3.1. Effects of Antibiotics Concentration and Type on C. pyrenoidosa

The response of microalgae to antibiotics varies with algal species, antibiotic class, and exposure level [13]. In this study, low mixed-antibiotic concentrations (<200 µg/mL per antibiotic) caused only modest inhibition, whereas high concentrations (>400 µg/mL per antibiotic) markedly restricted growth. Such a threshold-like response is consistent with previous reports showing that antibiotic toxicity in microalgae becomes much more pronounced once exposure surpasses the physiological buffering capacity of the cells [14]. This dose-dependent pattern may arise because cells can partially accommodate low-level exposure, whereas higher concentrations overwhelm cellular homeostasis. Antibiotics are known to induce oxidative stress in microalgae, and sublethal exposure can trigger protective responses such as antioxidant activity and metabolic adjustment [15]. However, these defenses are energetically costly and often coincide with reduced growth. When exposure intensifies, oxidative and metabolic damage can no longer be effectively compensated, resulting in stronger physiological inhibition [16].

The single-antibiotic experiment further showed that the inhibitory effects were highly antibiotic-specific. This result was expected because the tested compounds differ fundamentally in their primary modes of action: β-lactams such as ampicillin interfere with bacterial cell-wall synthesis [12]; sulfanilamide inhibits folate biosynthesis by competing with para-aminobenzoic acid [10]; chloramphenicol targets the 50S ribosomal subunit [12]; and streptomycin binds the 30S ribosomal subunit and disrupts protein synthesis [11]. In our study, AMPS was largely neutral, SA and CHL caused only mild or partly compensated effects, whereas STRS emerged as the dominant direct stressor to *C. pyrenoidosa*. This antibiotic-type specificity is important because it shows that not all antibiotics impair algal physiology to the same extent. Similar observations have been reported in *Haematococcus lacustris*, in which streptomycin greatly inhibited algal growth rather than functioning as a benign purification aid [17]. Such antibiotic-specific responses have also been reported in *Chaetoceros muelleri* [18] and *Chrysotila roscoffensis* [19], in which ampicillin exerted little effect on growth, whereas streptomycin significantly reduced cell density after prolonged cultivation.

The particularly strong effect of STRS is biologically reasonable in view of plastid evolution. Chloroplasts retain bacterial-type 70S ribosomes as a consequence of their endosymbiotic origin, and plastid translation is essential for the synthesis and maintenance of core photosynthetic components [20]. Structural studies have confirmed that the chloroplast translational apparatus is still based on a bacterial-type 70S ribosome, providing a mechanistic basis for the susceptibility of photosynthetic eukaryotes to ribosome-targeting antibiotics such as streptomycin [21]. In our study, STRS reduced Fv/Fm and light-use efficiency by 51.4% and 44.4%, respectively. Consistent with this observation, studies in *Chrysotila* roscoffensis have shown that Fv/Fm responds earlier and more sensitively than growth or biomass to antibiotic stress, indicating that photosynthetic impairment is an early indicator of antibiotic toxicity [19]. Under optimal conditions, Fv/Fm values of approximately 0.7 are typical of unstressed chlorophytes [22]. Therefore, STRS shifted *C. pyrenoidosa* from a growth-oriented photosynthetic state toward a stressed, photoprotective state. In line with this, previous studies in *Chlorella vulgaris* reported that streptomycin reduced growth, disturbed photosynthesis-related gene transcription, blocked electron transport, increased oxidative stress, and decreased both PSII photochemical activity and D1 protein content [23,24].

### 3.2. Antibiotic Stress Altered Pigment Homeostasis and Induced Lipid Remodeling

Photosynthetic pigments provide a sensitive readout of algal physiological status as they are linked to light harvesting, energy transfer, and photosynthesis [25]. In our study, the single-antibiotic experiment further showed that pigment responses were strongly antibiotic-specific. AMPS had little overall effect on pigment composition, whereas SA and CHL maintained or even enhanced total pigment content. By contrast, STRS caused the clearest pigment-damage phenotype, characterized by a sharp reduction in chlorophyll a and total pigment content. This distinction is important because it indicates that the observed pigment changes were not a generic response to all antibiotics but depended strongly on their mode of action.

Even when chlorophyll *a* declined, carotenoid levels were maintained or increased, particularly under CHL and STRS treatments. Carotenoids are important non-enzymatic antioxidants that quench excess excitation energy and reactive oxygen species generated under stress [26]. The increase in carotenoids under STRS, together with the rise in the carotenoid-to-chlorophyll ratio under stronger mixed-antibiotic exposure, therefore supports interpretation that antibiotic stress induced a photoprotective response and activated non-enzymatic ROS scavenging system rather than a simple uniform loss of pigments.

Meanwhile, in our study, chlorophyll *a* was strongly negatively correlated with PUFAs, particularly ALA, whereas carotenoids showed the opposite pattern. The inverse association between chlorophyll abundance and PUFA levels reinforces the link between photosynthetic impairment and lipid remodeling. This relationship suggests that pigment remodeling and fatty-acid remodeling were not independent phenomena. Rather, they formed a coordinated stress response program. One plausible explanation is that when photosynthesis is impaired, *C. pyrenoidosa* redirects metabolism toward membrane stabilization and stress protection. The enrichment of n-3 PUFA, especially ALA, may help preserve thylakoid membrane fluidity, facilitate protein mobility within crowded photosynthetic membranes, and support repair processes in damaged photosystems. Studies on chloroplast and thylakoid membranes likewise emphasize that polyunsaturated fatty acids, particularly 18:3 and 16:3, are major structural components of photosynthetic membranes and are important for membrane dynamics and PSII repair under stress [27].

At the same time, the rise in ALA may also reflect the activation of lipid-derived stress signaling. ALA is the precursor of jasmonate-related oxylipins, and its accumulation is frequently associated with abiotic stress responses and membrane-derived signaling processes [28]. Thus, the substantial increase in C18:3n3 under STRS may have dual significance: it may contribute structurally to membrane stabilization and function as a metabolic reservoir for downstream stress-signaling molecules. This possibility is especially relevant in light of our later metabolomics results, which revealed coordinated shifts in several oxidized lipid metabolites under STRS exposure. Therefore, rather than isolated endpoints, pigment alteration and lipid remodeling should be regarded as two tightly linked manifestations of stress-response strategy.

### 3.3. STRS Reshaped the Culture-Associated Microbiome in C. pyrenoidosa

*C. pyrenoidosa* exhibits a negative zeta potential, which facilitates the attachment of bacteria to its surface [29]. Symbiotic bacteria have been reported to enhance microalgal growth through various cooperative mechanisms, including gas exchange and compensation, secretion of specific phytohormones, nutrient acquisition, and signal exchange [30]. Direct experimental evidence has shown that bacteria can support algal growth through vitamin B12 provision in exchange for fixed carbon [31].

Our data showed that STRS did not merely alter the relative abundance of a few taxa but fundamentally changed bacterial community structure. At the phylum level, the community shifted from a comparatively balanced assemblage dominated by Proteobacteria and Bacteroidota to a strongly Proteobacteria-enriched state. Members of Proteobacteria are well known for their roles in nitrogen and phosphate metabolism and biodegradation. Proteobacteria commonly dominate wastewater-treatment systems and are strongly associated with *C. pyrenoidosa* cultures [32].

In our case, the most conspicuous class-level shift was the enrichment of Gammaproteobacteria and Alphaproteobacteria. Similar increases in these classes have been observed in microalgae exposed to stressors such as levofloxacin [32], nanoparticles [33], or other environmental pressures [34]. Both classes are recognized as microalgal-promoting bacteria [32,35]. Members of Alphaproteobacteria may promote algal growth and stabilization of microalgal–bacterial consortia, whereas members of Gammaproteobacteria are known for their capacity to degrade toxic and organic compounds [35,36]. Some Gammaproteobacteria also secrete extracellular polymeric substances (EPS), which can enhance their tolerance of environmental stress [37]. The family-level results showed that STRS favored lineages belonging to Rhizobiaceae and Pseudomonadaceae. One study reported that Rhizobiaceae includes nitrogen-fixing bacteria and may be associated with nitrogen cometabolism in microalgal-bacterial systems [38]. These findings indicate that STRS did not affect all bacterial taxa uniformly but selected for a restricted subset of bacterial lineages within the *C. pyrenoidosa* culture.

STRS exposure was accompanied by changes in both bacterial community composition and the lipid-related metabolite profile of *C. pyrenoidosa*. These bacterial changes coincided with reductions in most representative LysoPCs, glycerophosphocholine, and ALA-derived oxylipins under STRS exposure. Oxylipin profiles in microalgae are sensitive to environmental stress, suggesting that these associations may reflect coordinated changes in membrane lipid turnover and stress-responsive lipid metabolism [39]. A functional connection with the phycosphere is biologically plausible. Phycospheric bacterial volatiles can alter lipid productivity and fatty acid composition in Chlorella vulgaris [40]. Bidirectional exchange of iron and organic carbon has also been demonstrated between Chlorella sorokiniana and its bacterial partner [41]. The STRS-enriched taxa may therefore influence the chemical environment surrounding the algal cells, while changes in algal metabolites may also select for these bacteria. Nevertheless, Procrustes and Mantel analyses further demonstrated statistical covariation between bacterial community composition and the algal metabolite profile. However, these analyses do not establish causality, directionality, or a coordinated microalga–bacteria biological mechanism. STRS can directly affect susceptible bacteria while independently impairing the bacterial-type translation machinery of algal chloroplasts. Therefore, the bacterial-community and metabolomic alterations observed here are most conservatively interpreted as separate, parallel toxicological responses to the same STRS treatment. Whether particular bacterial taxa contribute to algal lipid remodeling would require further experiments using axenic cultures, xenic controls, and controlled re-inoculation with candidate taxa such as *Aminobacter*, *Pseudomonas*, or *Delftia*.

However, close interactions between microalgae and bacteria raise an important concern about the selection and spread of antibiotic-resistant bacteria and the potential horizontal transfer of antibiotic resistance genes (ARGs) under antibiotic exposure. For example, several studies have identified *Brevundimonas*, *Hydrogenophaga*, *Aquimonas*, and *Sphingopyxis* as potential ARG hosts [33,42,43,44,45]. In our study, none of these genera except *Hydrogenophaga* and *Brevundimonas* were detected. Whereas the relative abundances of both genera were indeed decreased after STRS exposure. Interestingly, *Pseudomonas*, which increased after STRS treatment, has been reported to correlate negatively with ARG abundance [46]. Despite this observation, whether STRS treatment promotes the gradual selection or accumulation of antibiotic resistance remains uncertain and requires further investigation.

### 3.4. Broader Significance, Methodological Implications, and Future Directions

Antibiotic-based purification is still the most commonly adopted route for bacterial removal from microalgae [9]. To broaden antibacterial coverage and improve decontamination efficiency, many studies have further relied on antibiotic cocktails that combine drugs with different targets [46]. Notably, network-based synthesis of the axenisation literature identified streptomycin as one of the most frequently used antibiotics, either alone or in combination, particularly together with penicillins or other aminoglycosides [8]. However, our results clearly show that STRS is not physiologically neutral for *C. pyrenoidosa* but instead acts as a strong stressor. Therefore, antibiotic choice should be treated as a species-specific decision rather than a routine procedural step, and sensitivity testing is essential to identify appropriate antibiotic types and concentrations.

To reduce reliance on antibiotics, a growing number of studies have explored alternative or combined strategies that emphasize physical separation or antibiotic-sparing workflows. For example, Doppler et al. (2021) reported a simple method using fluorescence-activated cell sorting to obtain axenic culture [47]. However, the effectiveness of such precision-based separation approaches is limited when bacteria are tightly attached to the algal surface or embedded in stable algal–bacterial aggregates. More recently, Iyer et al. (2025) investigated an antibiotic-free alternative based on anoxic treatment combined with Rose Bengal photosensitization, which successfully decontaminated several oleaginous microalgae and even outperformed conventional antibiotic treatment in some cases [34]. This suggests that future decontamination or axenisation strategies should move beyond routine antibiotic use and focus on simpler, cheaper, and more sustainable methods for different microalgal systems.

In our study, the mixed-antibiotic exposure markedly reduced culturable bacterial abundance, although detectable bacteria remained even at the highest concentration tested. Yet an important methodological question remains: even if an axenic culture is obtained in practice, can all bacteria truly be eliminated? As several reviews have pointed out, there is no single universally accepted definition of axenicity [8,9,48]. Due to close symbiotic relationships between microalgae and bacteria, some algae even grow poorly under axenic conditions because beneficial or obligate bacterial partners have been removed [8]. At the same time, confirming axenicity is itself technically challenging, since plate tests, microscopy, flow cytometry, and sequencing all have limits when used alone [49]. For example, an important limitation of this study is that residual bacterial viability was evaluated only by plate counting. Antibiotic exposure can induce a viable but nonculturable state, in which bacterial cells remain viable but fail to form colonies under routine cultivation conditions. Therefore, as Pokorny et al. (2022) stated, an “axenic culture” is best understood as an operational rather than absolute state, referring to a culture without detectable contaminants under the methods applied [48].

Taken together, there is still no universal method for establishing axenic microalgal cultures, and this problem is especially evident in green algae [8]. If conditions permit, future efforts should begin not with empirical antibiotic selection alone, but with profiling the associated bacterial community and evaluating algal sensitivity, so that antibiotic cocktails, concentrations, treatment duration, and recovery conditions can be designed on a more rational, species-specific basis. At the same time, axenization should not depend solely on antibiotics, instead, multi-step workflows that integrate targeted chemical treatment with physical separation and rigorous verification are likely to be more robust.

## 4. Materials and Methods

### 4.1. Algal Strain, Culture Conditions, and Experimental Design

*Chlorella pyrenoidosa* SHOU-1002 was originally isolated from wastewater collected from the Shanghai Xiangxin Dongtan Breeding Pig Farm (Shanghai, China) and is maintained at Shanghai Ocean University (Shanghai, China). The strain was previously identified based on morphological characteristics, internal transcribed spacer (ITS) sequencing, and phylogenetic analysis [27]. The alga was cultured in f/2 medium [50] supplemented with 1.5 g/L yeast extract (Shengsi Biochemical Technology, Shanghai, China) [51]. Cultures were maintained at 28 °C under continuous illumination at 70 µmol m^−2^ s^−1^. The initial algal cell density was adjusted to 2.39 × 10^6^ cells/mL. Culture flasks were manually shaken three times per day.

Ampicillin sodium (AMPS), sulfanilamide (SA), chloramphenicol (CHL), and streptomycin sulfate (STRS) were selected. The purity, catalog number, and stock solvent of each antibiotic are provided in Table S1. Two experiments were conducted to evaluate the effects of antibiotic concentration and antibiotic type. For the concentration-response experiment, the four antibiotics were mixed at equal concentrations. The nominal concentration of each antibiotic in the mixture was 0, 50, 100, 200, 400, or 800 µg/mL, corresponding to total antibiotic concentrations of 0, 200, 400, 800, 1600, and 3200 µg/mL, respectively. Unless otherwise stated, the concentrations reported for the mixed-antibiotic treatments refer to the concentration of each individual antibiotic. In the antibiotic-type experiment, each antibiotic was applied separately at 200 µg/mL, while an untreated culture without antibiotics served as the control (CK). This concentration was selected as an intermediate exposure level based on the concentration-response experiment, allowing antibiotic-specific physiological effects to be compared while avoiding the severe growth inhibition observed at higher concentrations. Each group consisted of 3 biological replicates.

Antibiotics remained in the culture medium throughout the five-day exposure period. All physiological measurements and samples for bacterial-community and metabolomic analyses were collected directly from cultures containing the assigned antibiotic treatment. The algal cells were not washed or transferred to fresh antibiotic-free medium before these measurements. Therefore, the experimental design evaluated responses during antibiotic exposure rather than recovery after antibiotic withdrawal.

### 4.2. Enumeration of Culturable Bacteria

The abundance of culturable bacteria in the algal cultures was determined by serial dilution and plate counting. Samples from each treatment were serially diluted with sterile physiological saline from 10^0^ to 10^−7^. An aliquot of 30 µL from each dilution was spread onto nutrient agar plates. The plates were incubated for 48 h at 28 °C under a 12 h light/12 h dark photoperiod and a light intensity of 70 µmol m^−2^ s^−1^. Plates containing 30–300 colonies were selected for counting. Bacterial abundance was calculated according to the dilution factor and expressed as colony-forming units per milliliter (CFU/mL). Plate counting was used to quantify bacteria capable of forming colonies under the specified cultivation conditions. This assay was not intended to determine total bacterial abundance or to exclude the presence of viable but nonculturable cells.

### 4.3. Determination of Algal Growth and Photosynthetic Parameters

Algal cell density was measured daily throughout the cultivation period. Samples were fixed with Lugol’s iodine solution, and cells were counted using a hemocytometer. The relative growth rate was calculated as: µ = (ln *N*_t_ − ln *N*_0_)/*t*, where *N*_0_ represents the initial algal cell density and *N*_t_ represents the cell density after cultivation for time *t*.

Photosynthetic performance was evaluated in the single-antibiotic experiment to compare the effects of different antibiotics on the photosynthetic system of *C. pyrenoidosa*, using a Phyto-PAM phytoplankton fluorometer (Walz, Germany). Prior to measurement, algal samples were dark-adapted for 5 min. Fv/Fm was used as an indicator of the maximum photochemical efficiency of PSII. The effective quantum yield of PSII [Y(II)], maximum electron transport rate (ETRmax), and initial slope of the rapid light curve (α), representing light-use efficiency, were also determined.

### 4.4. Determination of Pigment Content and Fatty-Acid Composition

Day 5 was selected as the sampling endpoint because it represented the completion of the predefined 5-day exposure period, by which time clear treatment-dependent physiological responses had developed. The same endpoint was used for downstream biochemical and microbial analyses to facilitate comparison across datasets. Chlorophyll a, chlorophyll b, and carotenoid contents were determined spectrophotometrically according to the method of Ritchie (2006) [52]. Fatty-acid composition was analyzed after direct transesterification of the algal biomass according to Griffiths et al. (2010) [53]. Fatty acid methyl esters (FAMEs) were separated using an Agilent 7890 gas chromatographic system (Agilent Technologies, Santa Clara, CA, USA) equipped with an SP-2560 fused-silica capillary column (100 m × 0.25 mm × 0.20 µm film thickness; Supelco, Darmstadt, Germany). Individual FAMEs were identified by comparison with authentic standards. The relative abundance of each fatty acid was expressed as a percentage of the total identified FAMEs.

### 4.5. Bacterial 16S rRNA Gene Sequencing and Community Analysis

At the end of the 5-day experiment, algal samples (*n* = 4 per group) were collected from the control (CK) and STRS-treated group. Total microbial DNA was extracted using the E.Z.N.A.^®^ Soil DNA Kit (Omega Bio-tek, Norcross, GA, USA), assessed by 1% agarose gel electrophoresis, and quantified using a NanoDrop 2000 spectrophotometer (Thermo Fisher Scientific, Waltham, MA, USA). The V3–V4 region of the bacterial 16S rRNA gene was amplified using barcoded primers 338F (5′-ACTCCTACGGGAGGCAGCAG-3′) and 806R (5′-GGACTACHVGGGTWTCTAAT-3′), TransStart FastPfu DNA Polymerase (TransGen Biotech, Beijing, China), and an ABI GeneAmp 9700 thermal cycler under the following conditions: initial denaturation at 95 °C for 3 min; 27 cycles of denaturation at 95 °C for 30 s, annealing at 55 °C for 30 s, and extension at 72 °C for 45 s; followed by final extension at 72 °C for 10 min and holding at 10 °C. Pooled amplicons were purified using the AxyPrep DNA Gel Extraction Kit (Axygen, Union City, CA, USA) and quantified using the QuantiFluor™-ST system (Promega, Madison, WI, USA). Libraries were prepared using the TruSeq™ DNA Sample Preparation Kit (Illumina, San Diego, CA, USA) and paired-end sequenced on an Illumina NextSeq 2000 platform by Majorbio Bio-Pharm Technology Co., Ltd. (Shanghai, China).

Raw reads were quality-filtered using fastp v0.19.6 and merged using FLASH v1.2.11. Reads containing ambiguous bases or shorter than 50 bp after quality trimming were removed. Paired-end reads were merged with a minimum overlap of 10 bp and a maximum mismatch ratio of 0.2. The resulting sequences were clustered into operational taxonomic units (OTUs) at 97% sequence similarity using UPARSE v7.1, with chimeric sequences removed during clustering [54,55]. Taxonomic assignment was performed using the RDP Classifier v2.11 against the SILVA 16S rRNA database (release 138) [56], with a confidence threshold of 70%. The OTU table was rarefied to the lowest sequencing depth among samples before diversity analysis.

Good’s coverage, Shannon index, and Simpson dominance index were calculated using Mothur v1.30.1 [57]. Differences in community structure were examined by principal coordinate analysis (PCoA) based on Bray–Curtis dissimilarities at the OTU level. Taxonomic composition was summarized at different classification levels. Differentially abundant bacterial lineages were identified using linear discriminant analysis effect size (LEfSe) [58], with *p* < 0.05 and an LDA score > 2. Differences in taxon abundance between CK and STRS were further evaluated using the Wilcoxon rank-sum test. Bioinformatic and statistical analyses were conducted on the Majorbio Cloud platform (https://cloud.majorbio.com).

### 4.6. Untargeted Metabolomics and Lipid-Focused Analysis

Algal samples (*n* = 4) were collected on day 5, and 50 mg of each sample was extracted with 400 µL methanol/water (4:1, *v*/*v*) containing 0.02 mg/mL L-2-chlorophenylalanine. Samples were homogenized at −10 °C for 6 min, sonicated at 5 °C for 30 min, and centrifuged at 13,000× *g* for 15 min at 4 °C. The supernatants were analyzed using a Thermo UHPLC-Q Exactive HF-X system coupled to an ACQUITY UPLC HSS T3 column (100 × 2.1 mm, 1.8 µm). The mobile phases were water/acetonitrile (95:5, *v*/*v*) containing 0.1% formic acid (A) and acetonitrile/isopropanol/water (47.5:47.5:5, *v*/*v*/*v*) containing 0.1% formic acid (B). The gradient is provided in Supplementary Table S1. The injection volume was 2 µL, and the column temperature was 40 °C. Mass spectra were acquired in positive and negative ion modes over *m*/*z* 70–1050. The full-MS and MS/MS resolutions were 60,000 and 7500, respectively.

Raw data were processed using Progenesis QI (Waters Corporation, Milford, MA, USA) for peak detection, retention-time alignment, and normalization. Metabolites were annotated by matching accurate mass and MS/MS spectra against HMDB and METLIN, with a mass tolerance of <10 ppm. Putative lipid-related metabolites were identified as the union of compounds classified as “Lipids and lipid-like molecules” in HMDB and those assigned to lipid metabolism pathways in KEGG. Duplicates were removed before subsequent analysis. Univariate comparisons between the CK and STRS groups were performed using two-sided unpaired Student’s t-tests. The resulting *p*-values were adjusted for multiple comparisons using the Benjamini–Hochberg procedure. Differential metabolites were defined as those with VIP_pred_OPLS-DA > 1 and FDR-adjusted *p*-value < 0.05. Representative differential lipids were visualized by hierarchical clustering after Z-score normalization. KEGG enrichment and topology analyses were conducted using KEGG v94.2. Enrichment *p* values were adjusted using the Benjamini–Hochberg method, and pathway impact was calculated using relative-betweenness centrality.

### 4.7. Integrated Microbiome–Metabolome Analysis

Data from matched algal samples were integrated to examine associations between bacterial community composition and metabolite profiles. Across the CK and STRS samples, genus-level bacterial and metabolite profiles were ordinated by PCoA using Bray–Curtis and Euclidean distances, respectively, and their concordance was evaluated by Procrustes analysis. Mantel tests using Spearman correlations were then performed to assess associations between the Bray–Curtis distance matrices of selected bacterial classes or genera and those of the 15 most strongly differential lipid-related metabolites.

### 4.8. Statistical Analysis

Data are presented as means ± standard deviations (SD). Statistical analyses were performed using IBM SPSS Statistics 26. Normality was assessed using the Shapiro–Wilk test, and homogeneity of variance was evaluated using Levene’s test. For endpoint variables in the mixed-antibiotic concentration experiment, differences among concentration groups were evaluated by one-way ANOVA followed by Duncan’s multiple range test. For the individual-antibiotic experiment, endpoint variables were analyzed by one-way ANOVA followed by Dunnett’s multiple-comparison test, with the untreated CK group as the reference. Time-course data, including cell density, Fv/Fm, Y(II), α, and ETRmax, were analyzed using two-way repeated-measures ANOVA, with treatment as the between-subject factor and cultivation time as the within-subject factor. Mauchly’s test was used to assess sphericity, and the Greenhouse–Geisser correction was applied when necessary. Following a significant treatment × time interaction, simple comparisons were performed at individual sampling times using the multiple-comparison procedure appropriate to the experiment. Differences were considered statistically significant at *p* < 0.05.

The concentration causing 50% inhibition of algal growth (IC50) was estimated from growth-inhibition data in 72 h by fitting a four-parameter log-logistic concentration–response model in GraphPad Prism (version 11). Growth inhibition was calculated relative to the antibiotic-free control. The IC_50_ was expressed on a per-antibiotic basis and reported with a 95% confidence interval. Pearson correlation analysis was used to assess associations between pigment contents and fatty-acid composition.

## 5. Conclusions

This study demonstrates that continuous antibiotic exposure produces concentration- and compound-specific effects on *Chlorella pyrenoidosa* and its culture-associated bacteria. It is a dose- and antibiotic-specific disturbance that affects both the algal host and its associated bacterial community. High exposure progressively inhibited algal growth and photosynthesis, disrupted pigment homeostasis, and altered fatty-acid composition. The overall physiological disturbance caused by the tested antibiotics generally increased in the order ampicillin sodium < sulfanilamide < chloramphenicol < streptomycin sulfate. Among them, streptomycin sulfate (STRS) exerted the strongest effects, reducing growth, with 24.18% lower cell density and 17.39% lower relative growth rate; impairing photosynthetic performance, with 51.4% lower Fv/Fm and 44.4% lower light-use efficiency; reducing pigment contents, with 15.31% lower total pigment content and 34.70% lower chlorophyll *a* content; and altering fatty-acid composition, with 33.33% higher total PUFA and 135.0% higher ALA proportions. STRS also altered the culture-associated bacterial community, with a pronounced shift toward Proteobacteria dominance from 44.54% to 90.66%, characterized by an abundance of *Aminobacter*, *Pseudomonas*, and *Delftia*. FDR-corrected metabolomic analysis identified 165 differentially abundant lipid-related metabolites, including 66 increased and 99 decreased metabolites, with α-linolenic acid metabolism and glycerophospholipid metabolism among the most strongly affected pathways. Procrustes and Mantel analyses further showed that bacterial community restructuring statistical covaried with changes in the lipid-related metabolome, suggesting parallel toxicological responses to STRS. This work shows that antibiotic treatment should be viewed as a selective physiological and ecological perturbation rather than a simple bacterial-removal step. Microalgal axenization should therefore be based on species-specific sensitivity testing and rational selection of antibiotic type and concentration.

## Figures and Tables

**Table 1:** Effects of mixed antibiotic on the relative growth rate of *C. pyrenoidosa*.

Group	MIX-0	MIX-50	MIX-100	MIX-200	MIX-400	MIX-800
Relative growth rate (µ)	0.39 ± 0.01	0.34 ± 0.02	0.33 ± 0.03	0.32 ± 0.01	0.28 ± 0.02	0.19 ± 0.02

**Table 2:** Effects of individual antibiotics on the relative growth rate of *C. pyrenoidosa*.

Group	CK	AMPS	SA	CHL	STRS
Relative growth rate (µ)	0.46 ± 0.01	0.47 ± 0.00	0.44 ± 0.01	0.46 ± 0.01	0.38 ± 0.00

## Data Availability

The original contributions presented in this study are included in the article/Supplementary Materials. Further inquiries can be directed to the corresponding author(s).

## Supplementary Materials

The following supporting information can be downloaded at: https://www.mdpi.com/article/10.3390/ijms27188325/s1.

## Abbreviations

The following abbreviations are used in this manuscript:

ALA	α-linolenic acid
AMPS	ampicillin sodium
CFU	colony-forming unit
CHL	chloramphenicol
CK	untreated control
FAME	fatty acid methyl ester
FLASH	fast length adjustment of short reads
ITS	internal transcribed spacer
LDA	linear discriminant analysis
LEfSe	linear discriminant analysis effect size
LysoPC	lysophosphatidylcholine
MIX	mixed-antibiotic treatment
MS/MS	tandem mass spectrometry
OTU	operational taxonomic unit
PAM	pulse-amplitude modulation
PSII	Photosystem II
RDP	Ribosomal Database Project
ROS	reactive oxygen species
SA	sulfanilamide
STRS	streptomycin sulfate
VIP	variable importance in projection
